# Unpacking the role of financial literacy in the debt-mental health nexus: evidence from China

**DOI:** 10.3389/fpubh.2025.1563297

**Published:** 2025-05-07

**Authors:** Yunchao Cai

**Affiliations:** School of Economics and Management, Ankang University, Ankang, Shaanxi, China

**Keywords:** household indebtedness, mental health, financial literacy, non-housing debt, China

## Abstract

**Introduction:**

Despite the growing challenges associated with household debt, research on factors influencing its relationship with psychological well-being remains limited. This study investigates the role of financial literacy in the nexus between household indebtedness and mental health, addressing a significant gap in the literature.

**Methods:**

Using data from the China Family Panel Studies (CFPS) 2014 wave, a nationally representative dataset, we analyze how financial literacy interacts with household debt and mental health outcomes. Multiple model specifications are employed to assess the moderating and mediating effects of financial literacy.

**Results:**

Our findings reveal two key roles of financial literacy: (1) it improves mental health by reducing household indebtedness, and (2) it moderates the negative relationship between debt and mental health. Notably, basic financial literacy is a critical factor, particularly in explaining the effects of non-housing debt (as opposed to housing debt).

**Discussion:**

The study highlights the dual function of financial literacy in mitigating the adverse psychological effects of household debt. Policymakers and financial educators should consider promoting financial literacy as a tool to enhance mental health, especially in contexts of high indebtedness. Future research could explore additional mediators and cultural variations in this relationship.

## 1 Introduction

The rapid growth of household debt has emerged as a prevalent global phenomenon over the past two decades (1). Many households have benefited from borrowing to finance major expenses, manage cash flow, and improve investment opportunities, thereby enhancing their overall wellbeing. However, existing empirical research indicates that the rise in household debt has also introduced new challenges. These challenges can negatively affect household wellbeing, particularly regarding psychological wellness (2–4).

To date, numerous studies in the field of psychology provide evidence suggesting that higher levels of household indebtedness can lead to mental health issues, such as depression (5, 6), anxiety (7, 8), and other mental disorders (9, 10). Explanations for this association suggest that individuals with higher levels of indebtedness are more likely to experience financial distress (11) or to worry about their financial situations (12), which can adversely impact their mental wellbeing. This impact also appears to intensify during periods of increasing potential for external shocks (13).

Based on the extensive evidence highlighting the significant link between household indebtedness and psychological wellbeing, scholars have made efforts to propose strategies for mitigating these effects. One prominent recommendation put forth by many researchers is to reduce the level of leverage. This can be achieved by either decreasing the amount of debt or increasing the quantity of buffering assets. In addition, empirical evidence suggests that maintaining a smaller proportion of unsecured debts could be advantageous as these debts tend to have greater implications compared to secured debts (14). A third avenue of suggestion that has received significantly less attention involves improving households’ financial literacy (15). Research on financial literacy indicates that enhancing one’s financial knowledge and skills may serve as a more effective strategy, as individuals equipped with such knowledge are better able to manage their finances, particularly in relation to debt decisions and debt management (16). However, despite the importance of this topic, limited studies have explicitly examined the role of financial literacy as a potential factor in the relationship between household debt and mental health issues. Given that the objective of all credit stakeholders is to maximize benefits and minimize negative outcomes, recommending solely the reduction of debt or imposing constraints on debt expansion may prove challenging within the current financial system. Further research is needed to investigate how financial literacy might contribute to addressing this issue.

Therefore, this study aims to investigate the role of financial literacy in the relationship between household debt and mental health. The investigation will analyze this mechanism in two ways, considering two potential processes in which financial literacy may be involved. First, we treat financial literacy as an explanatory factor for household indebtedness and examine its impact on psychological wellbeing through household debt. This process considers household indebtedness as a mediating variable, elucidating how financial literacy influences mental health via its effects on household debt. The objective is to validate the hypothesis found in existing literature, which posits that higher levels of financial literacy can help reduce household indebtedness, thereby decreasing the likelihood of mental health issues.

Second, we explore the potential moderating role of financial literacy in the relationship between household debt and mental health. In this context, financial literacy is considered a moderating factor, and we investigate whether individuals with higher levels of financial literacy are better equipped to mitigate the impact of household indebtedness on mental health. This investigation aims to provide valuable insights into the potential protective role that financial literacy may play, particularly regarding mental health outcomes.

Our investigation may help to inform the development of targeted interventions and educational programs designed to promote financial literacy and its positive effects on mental health outcomes. Since discussions regarding factors that may mitigate the impact of household debt on subjective wellbeing are still in their early stages, identifying factors that can reduce or eliminate the negative relationship between debt and mental health is crucial. This is significant not only for financial institutions concerned with the social value of their products but also for the overall wellbeing of society, particularly as an increasing number of households face indebtedness in many countries (17).

The study is structured into five sections. Section 1 presents the introduction of the study. Section 2 reviews the relevant literature and discusses theoretical concepts regarding the relationship between household debt and mental health, financial literacy, and economic outcomes, as well as the role of financial literacy in buffering the stress process. Section 3 outlines the study’s methodology, followed by the presentation of results in Section 4. The final section discusses the findings and offers suggestions for further research.

## 2 Literature review

### 2.1 Household debt and mental health

The investigations into the implications of household debt within economic studies primarily focus on the link between household debt and economic wellbeing (18, 19). However, the potential effects of the “pain of paying” debt, which can undermine the pleasure derived from consumption and lead to a loss of utility, have received relatively less attention from economists. When consumers overextend their credit to achieve a higher level of consumption, it is challenging to assert that the impact of debt is confined solely to their economic wellbeing. Psychologists, on the other hand, have extensively examined the psychological impacts of household debt. A significant and robust negative relationship between debt and mental health has consistently been observed in numerous studies, encompassing problems such as depression, anxiety, and other mental disorders (14, 20).

The stress process model is a prominent psychological framework designed to explain the multifaceted effects of various stressors on an individual’s mental wellbeing (21, 22). This model posits that stress is a dynamic process involving multiple stages and factors. Drentea and Reynolds (8) adapted the stress process model to illustrate several ways in which debt may relate to mental health. The first relationship proposed in their study is the direct effect of debt on mental health outcomes, such as symptoms of depression and anxiety. In this relationship, debt can be conceptualized as a daily stressor that gradually erodes an individual’s mental health. The second relationship suggests debt indirectly impacts mental health by reducing mastery and coping capacity, or through strain. Carrying debt can undermine one’s sense of mastery as individuals may feel embarrassed or inadequate about their inability to manage their financial wellbeing effectively. The third possible role of debt investigated by Drentea and Reynolds (8) is that debt may serve as a buffer to address immediate financial needs. In this context, debt can act as a financial resource that does not directly cause mental health problems but may help alleviate mental stress. However, their study of Miami-Dade residents only confirmed debt’s direct negative association with mental health not the other two.

In addition, Tay et al. (23) also recommended a conceptual model for understanding the relationship between debt and overall subjective wellbeing. Their model depicted two perspectives on how debt influences overall wellbeing. The first perspective is the bottom-up spill-over perspective, which suggests that subjective debt burden, rather than objective debt burden, may more significantly affect financial subjective wellbeing. This spill-over effect can extend to other life domains such as psychological wellbeing, leisure activities, marital satisfaction, and more. This is because financial wellbeing is a key aspect of life that can impact other areas. Constraints in financial situations may limit opportunities and benefits across various domains. The second perspective is the resource perspective, where debt is seen as a strain. Depleting resources, such as through accumulating debt, creates uncertainty and stress. This depletion can lower an individual’s subjective wellbeing as people strive to maintain and protect their resources (24).

Moreover, other studies also identified various factors that may mediate or moderate the relationship between household debts on subjective wellbeing. For instance, Jessop et al. (25) found that financial concern mediates the relationship between debt and health. Similarly, financial worry mediates the relationship between debt and life satisfaction (23). Gathergood (26) utilizing UK panel data discovered that the effect of debt payment problems on psychological wellbeing is influenced by social norms.

In terms of moderating factors, Tsai et al. (27) revealed that financial support from family members partially mitigates the negative effects of debt among a sample from Taiwan. Conversely, reliance on financial assistance from friends and banks may create a debt trap, leading to lower levels of life satisfaction and negative self-concept. Xiao et al. (28) examining data from China investigated the association between debt holding and subjective wellbeing, suggesting that borrowing sources and income levels moderate the relationship between debt holding and life satisfaction. Similarly, Gao et al. (3) focusing on older adults in China explored the moderating roles of social networks and expected support in the association between household financial indebtedness and depressive symptoms. They found that higher levels of household debt correlate with increased depressive symptoms among older adult individuals, but the presence of a supportive social network and expectations of help played a moderating role in this connection.

Finally, unlike the predominant focus on the negative impacts of household debt on wellbeing, debt can also serve instrumental purposes that yield positive effects. For example, student loans, used to cover the high costs of education, can provide students with opportunities for future improvement and personal growth. Prolonged debt repayment can function as a financial resource, offering stress-buffering effects that might mitigate the burden that would arise from large initial payments (23). Furthermore, high levels of consumer debt can enhance symbolic capital, providing sources of social prestige and status (29).

### 2.2 Financial literacy and economic outcomes

The world’s current financial environment is dynamic and increasingly complex, requiring individuals to have the ability to process information and make well-informed financial decisions. Neoclassical economic theory assumes that individuals make decisions based on complete information regarding costs and benefits. However, empirical evidence indicates that people exhibit bounded rationality (30), meaning their decision-making is constrained by cognitive limitations and imperfect information. A lack of financial knowledge or ability can hinder individuals from making decisions that would optimize their utility.

Research consistently demonstrates that financial literacy is critical in shaping household economic behaviors. Higher levels of financial literacy are associated with better personal financial management (31), improved investment behavior (32), increased adoption of appropriate financial services (33), and more effective retirement planning (34–36). Furthermore, financial literacy may enhance decision-making concerning assets, debts, and savings (37) and is linked to elevated levels of financial wellbeing (38). More importantly, studies also reveal that individuals with higher financial literacy are less likely to be effected with negative income shocks (16, 39). This resilience is due to their enhanced ability to assess and manage financial risks, as well as their proficiency in navigating complex financial landscapes.

In terms of household debt in particular, empirical evidence shows that a strong understanding of financial concepts is associated with more responsible debt behaviors. For instance, Lusardi and Tufano (15) found that individuals with lower levels of debt literacy tended to engage in financial transactions with higher fees. Financial literacy also empowers households to negotiate favorable terms for loans and financial products, thus potentially improving their overall financial situation (40). In addition, research also indicates that lower financial literacy is correlated with higher mortgage delinquency rates (41, 42). Stango and Zinman (43) found that those who struggle with accurately calculating interest rates from a stream of payments tend to borrow larger sums of money and accumulate less wealth.

Moreover, it is important to note that there is a difference in the way researchers define financial literacy (44). The measurement of financial literacy varies from the most famous “Big Three” measure to the most extensive definition by the OECD (45).^1^ In different studies, researchers have applied different dimensions of financial literacy individually or in combination. Some researchers only measured based on financial knowledge, since they believe that an increase in financial knowledge naturally leads to improved financial management practices, while some others consider individuals to be financially illiterate if they are unable to apply their financial knowledge to make informed decisions (46). Some consider a person with a higher level of financial literacy generally should demonstrate appropriate knowledge, attitude, and behavior in managing their personal finances (47).

Despite the variety of measuring financial literacy provides broader understanding on its concept, whereas these differences also lead to different results on the effects of financial outcomes. For example, French and McKillop (48) found that the component of money management skills are important determinants of the debt-to-income level and the possibility to borrow from high-cost lenders but not numerical skills. On the other hand, numerical ability is negatively related to delinquency and default (42). Morgan and Long (49) provided evidence that not all three components of financial literacy (financial knowledge, financial behavior, and financial attitude) are positively associated with financial inclusion, and it varies among different indicators of financial inclusion.

### 2.3 The stress buffering

Given the discussion on the relationship between household debt and mental health above, it is evident that understanding the factors that act as buffers between household debt and mental health is still lacking. The stress process model suggests that stressors can impact an individual’s wellbeing through a series of processes. Therefore, the stress-buffering model (21), which is based on the stress process paradigm, considers that coping resources can modify the connection between stressors and overall wellbeing by influencing how an individual perceives, evaluates, and handles potential stressors.

From a psychological perspective, Pearlin (21) suggests that stress can be associated with mental health either independently or influenced by pre-existing advantages and disadvantages linked to various factors. Evidence shows that factors such as self-efficacy (50), optimism (51), social networks (27), and socio-economic variables (4) may play a moderating role in the effect of financial distress on mental health. In addition to psychological factors, financial resources, such as assets, availability of credit options, savings, and other resources, may also provide stress-buffering effects by allowing individuals to outsource their financial commitments. The presence of debt can erode these financial resources, resulting in fewer buffering effects.

In addition to the psychological factors and financial resources, the knowledge or competency such as financial literacy may also function as types of coping resources that can affect the link between household debt and mental health. The possibility may operate through two ways. One is from the “resource perspective” mentioned above by Tay et al. (23). It is possible that when individuals have better knowledge or skills to effectively manage their finances, they may face fewer difficulties in budgeting, managing debt, and making informed financial decisions. This improved financial knowledge or positive financial behavior, leading to a better financial situation, can contribute to better financial wellbeing for individuals or households (38), which, in turn, contributes to better mental health. Second is from a stress model perspective; it is also possible that individuals who are financially literate may feel more confident and positive about their ability to manage their debt, thereby leading to less stress and better mental health, such as the concept of a scarcity mindset posits that financial strain imposes a cognitive burden, disrupting fundamental cognitive processes (52). Therefore, a higher level of financial literacy has the potential to alleviate this cognitive burden.

So far what we know is Ishii et al. (53) assessed financial literacy using Financial Knowledge Scale and find that the relationship between subjective socioeconomic and depression is weaker among individuals with high financial literacy than among individuals with low financial literacy. Mutlu and Özer (54) measured financial literacy based on subjective understanding of inflation, money management, and financial products. They provided evidence on financial literacy which moderates the relationship between internal locus of control and financial behavior of individual investors. Xiao and Xin (55) argue that the homo sociologicus index of financial literacy is a better predictor of happiness compared to the homo economicus index of financial literacy. However, in contrast, Xu and Sun (56) using two nationally representative datasets from China find that inclusive finance increased residents’ investment participation while decreasing their sense of happiness simultaneously.

**Figure 1:**
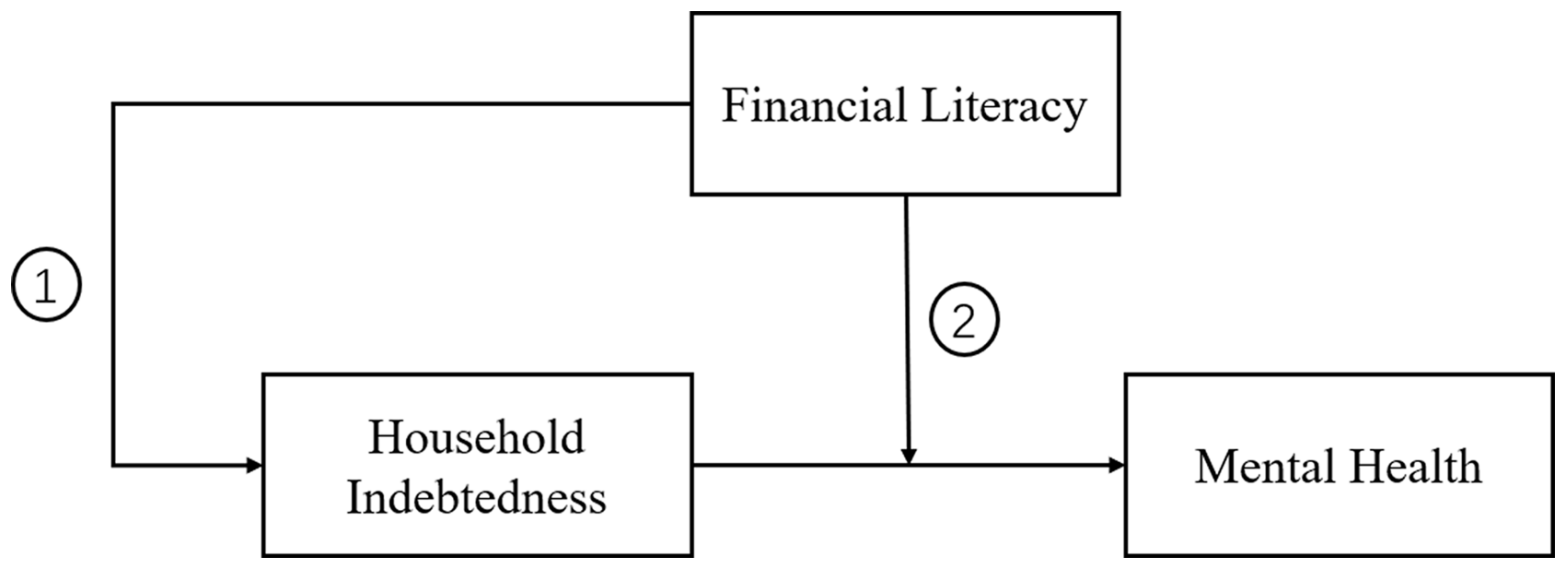
Interaction of financial literacy in the relationship between household debt and mental health.

Therefore, further research is needed to examine the factors that may influence the relationship between household debt and mental health. Based on the preceding discussion, this study hypothesizes that an individual’s level of financial literacy will play a significant role in the negative relationship between household debt and mental health in two ways (Figure 1). Consequently, we propose the following hypotheses:

*Hypothesis one: Higher levels of financial literacy are associated with lower levels of household indebtedness and, consequently, a reduced likelihood of experiencing mental health problems.*

*Hypothesis two: The inverse relationship between household indebtedness and mental health problems will be weaker for individuals with higher level of financial literacy.*

## 3 Method

### 3.1 Data

This study utilizes data from the China Family Panel Studies (CFPS) 2014 conducted by the Institution of Social Science Survey at Peking University China. The CFPS is a nationally representative survey conducted every 2 years since 2010 to gather socioeconomic information on Chinese households. The latest released wave is wave seven CFPS 2020. The CFPS 2014, which is the third wave of the survey, includes data from 13,946 households and 37,147 individuals across 29 provinces in China. While some waves of CFPS included some questions related to financial literacy, the 2014 wave is the only one that contains detailed information on thirteen financial literacy questions, ranging from various dimensions of financial knowledge. The financial literacy questions are only asked to the adult member that is most knowledgeable about the financial situation of the household. As a result, only 3,308 observations can be used in our analysis.

### 3.2 Measures

*Mental health*, which serves as the main dependent variable, was determined based on six mental status questions asked in the CFPS 2014. These questions range from *“How often in the past month did you feel so depressed that nothing could cheer you up?”* to questions such as *“How often in the past month did you feel that life was meaningless?”* (Appendix A). The responses to these questions were measured on a five-point scale, ranging from *Almost daily (1)* to *Never (5)*. By summing the scores from these six questions, a higher score indicates a better mental health status for the respondent.

#### 3.2.1 Household indebtedness

In the CFPS 2014 survey, respondents were asked to provide information on their household debt amount for various purposes and from different sources. This includes the total amount of the mortgage (including the interest) that has not been repaid yet. The total amount of loans owed to individuals and institutions (e.g., private loan institutions) other than banks that use for housing or innovations. The amount of bank loans (excluding mortgages). The amount of loans from non-banking sources and not used for housing or renovations. In this study, the household indebtedness is measured by calculating the overall household debt which include both bank and non-bank debts. Using this, we generated a debt-to-asset ratio with total asset provided for each household and categorized them into six groups^2^. The higher percentage of the debt-to-asset ratio indicates a higher level of indebtedness of the household. In addition, we calculated the housing loan-to-asset ratio and non-housing loan-to-asset ratio and generated ranges for these debt ratios.

#### 3.2.2 Financial literacy

The financial literacy is measured based on thirteen questions from CFPS 2014 that are able to specifically assess the level of financial knowledge of each respondent. The specific wording of all thirteen questions is provided in Appendix B. Consistent with the scoring system used in many previous studies, a correct answer is assigned a score of 1, while incorrect answers and “do not know/refuse” responses receive a score of 0. The financial literacy score is determined by the number of correct answers out of these thirteen questions, with scores ranging from a minimum of 0 to a maximum of 13. A higher score indicates a higher level of financial literacy.

In addition, similar to Niu et al. (57) and Wang et al. (58), we created both a basic financial knowledge index, consisting of five questions, and an advanced financial knowledge index, comprising eight questions. The difficulty level of the thirteen questions was taken into account when categorizing them. Questions pertaining to interest rates, compounding, inflation, and numeracy were classified as basic financial questions, while those involving the stock market, banking system, and mutual funds were considered advanced financial questions. Same as above, the basic and advanced financial literacy score is determined by the number of correct answers. A higher score indicates a higher level of basic financial literacy level or advanced financial literacy level.

#### 3.2.3 Control variables

Consistent with majority of literature that study on subjective wellbeing, the control variables included are gender (1—male; 0—female), age (age in year), education (—below primary; 1—primary; 2—middle school; 3—high school; 4—college and above), employed status (1—employed; 0—not employed), marriage status (1—married; 0—not married), urban (1—urban; 0—rural), household income (household annual income in ln), and self-evaluated health status (1—excellent; 2—very good; 3—good; 4—fair; 5—poor).

### 3.3 Research model

To achieve the objective of this research by investigating the two hypotheses proposed above, multiple regressions using robust ordinary least squares (OLS) regression models were applied in two ways. For the first hypothesis, which examines the mediating role of household indebtedness between financial literacy and mental health, two equations were conducted.


(1) $$Debt_{i}=\alpha_{0}+\alpha_{1}FL_{i}+\alpha_{2}X_{i}+\mu_{i}$$

(2) $$MH_{i}=\theta_{0}+\theta_{1}Debt_{i}+\theta_{2}FL_{i}+\theta_{3}X_{i}+\varepsilon_{i}$$

where $Debt_{t}$ represents different household indebtedness variables for respondent *i*. $FL_{i}$ represents different financial literacy variables for respondent *i*. $MH_{i}$ represents mental health for respondent *i*. $X_{i}$ is a vector of control variables. $\mu_{i}$ and $\varepsilon_{i}$ are the error terms in two equations. In Equation 1, we first examine the direct relationship between financial literacy and household indebtedness. Then, in Equation 2, we examine the direct effects of household indebtedness and financial literacy on mental health, with mental health as the dependent variable.

For the second hypothesis, which examines the moderating role of financial literacy between household indebtedness and mental health, the following equation is presented:


(3) $$MH_{i}=\beta_{0}+\beta_{1}Debt_{i}+\beta_{2}FL_{i}+\beta_{3}FL_{i}\times Debt_{i}+\beta_{4}X_{i}+\gamma_{i}$$

In Equation 3, the moderating role of financial literacy will hold when $\beta_{3}$ is significantly different from zero. Since the expected relationship between household indebtedness and mental health is negative, when $\beta_{3}$ is positive, it means the level of financial literacy plays a role in reducing the effect of household indebtedness on mental health. If $\beta_{3}$ is negative, the opposite moderating effect may hold.

## 4 Results

### 4.1 Descriptive results

Descriptive statistics for all the variables are presented in Table 1. In the sample, approximately 46% of participants are male. 86% of the respondents are married. Although only 61% of respondents are currently employed, 93% of them are from urban areas. The average mental health status is high (mean = 27.04, on a scale of 6–30), as is the health status (mean = 3.04, on a scale of 1–5). As the debt-to-asset ratio has been classified into six groups, the mean value of 1.69 suggests that a significant proportion of respondents (71.5%) reported having no debt. The average score for financial literacy, based on thirteen questions, is 6.18. This indicates that, on average, each respondent was unable to answer more than half of the questions correctly. When it comes to basic and advanced financial literacy, the average score for basic financial literacy (2.93 out of 5) is relatively higher compared to advanced financial literacy (3.35 out of 8).

**Table 1:** Descriptive statistics (observation = 3,308).

Variables	Mean	Std. Dev.	Min	Max
Mental health status	27.03	3.74	6	30
Financial literacy	6.18	2.97	0	13
Basic financial literacy	2.93	1.54	0	5
Advanced financial literacy	3.25	1.86	0	8
Debt-to-asset ratio (ranges)	1.69	1.29	1	6
Age	48.30	14.73	18	89
Male	0.46	0.50	0	1
Education level	2.43	1.21	0	4
Married	0.86	0.34	0	1
Employed	0.61	0.49	0	1
Urban	0.93	0.25	0	1
Household income (ln)	10.84	0.99	2.40	15.22
Health status	3.04	1.09	1	5

Moreover, in Table 2, we present the distribution of correct responses for the three financial literacy measures. The overall financial literacy scores approximate a normal distribution. While only a small percentage (3%) answered none of the 13 questions correctly and 1% achieved a perfect score, the most frequently occurring score was seven correct answers, representing 13% of the sample (mode).

**Table 2:** Distribution of correct answers for the three financial literacy measures.

Number of correct answers	Basic financial literacy (5 questions)	Advanced financial literacy (8 questions)	Financial literacy (All 13 questions)
0 questions	9.4%	5.4%	3.0%
1	11.5%	12.9%	4.3%
2	15.2%	19.6%	5.6%
3	21.6%	20.3%	7.2%
4	25.4%	17.5%	8.7%
5	16.8%	11.1%	11.4%
6		8.1%	12.6%
7		3.7%	13.0%
8		1.5%	11.9%
9			8.7%
10			6.1%
11			4.7%
12			2.0%
13			1.0%
Total	100%	100%	100%

In contrast, the distributions of correct answers for basic and advanced financial literacy exhibited distinct patterns. For basic financial literacy, respondents generally demonstrated a higher level of understanding. A total of 25.4% of respondents correctly answered four out of five questions, constituting the peak of the distribution. The percentage of respondents getting 0, 1, 2, 3, and 5 questions correctly are 9.4, 11.5, 15.2, 21.6, and 16.8%, respectively. These results suggest a relatively strong grasp of fundamental financial concepts within the surveyed population.

The pattern observed in advanced financial literacy starkly contrasts with that of basic literacy. The distribution is skewed toward lower scores, with the highest proportion of respondents answering only three out of eight questions correctly (20.3%). A decline in percentage is observed as respondents answer questions correctly, and 17.5, 11.1, 8.1, 3.7, and 1.5% of the respondents answered 4, 5, 6, 7, and 8 questions correctly. This indicates potential challenges in comprehending more complex financial instruments and concepts.

### 4.2 Results for hypothesis one

In Table 3, we present the results of the regression analysis aimed at examining the mediating role of household indebtedness in the relationship between financial literacy and mental health. The result in column (1) indicates a significant negative relationship between financial literacy and the household debt-to-asset ratio when covariates are controlled. In column (2), the result shows that the household debt-to-asset ratio is also significantly negatively related to mental health when same covariates are controlled. These findings suggest that the hypothesis one is supported, as financial literacy is positively associated with mental health through the mediating effect of household indebtedness, based on the data collected from Chinese households, but no direct relationship between financial literacy and mental health demonstrated in column (2). This indicates that respondents with a higher level of financial literacy tend to have a lower level of household indebtedness and will possibly have a higher level of mental health.

**Table 3:** Mediation analysis: debt-to-asset ratio as a mediator.

Variables	(1)	(2)	(3)	(4)	(5)	(6)
	Debt-to-asset ratio	Mental health	Debt-to-asset ratio	Mental health	Debt-to-asset ratio	Mental health
Debt-to-asset ratio		−0.280***		−0.273***		−0.284***
		(0.0576)		(0.0574)		(0.0577)
FL	−0.0335***	0.0335				
	(0.00917)	(0.0248)				
FL_basic			−0.0829***	0.116**		
			(0.0164)	(0.0453)		
FL_adv					−0.0183	−0.00652
					(0.0142)	(0.0372)
Controls	Yes	Yes	Yes	Yes	Yes	Yes
Observations	3,308	3,308	3,308	3,308	3,308	3,308
R-squared	0.046	0.110	0.049	0.111	0.042	0.109

FL represents financial literacy; FL_basic represents basic financial literacy; FL_adv represents advanced financial literacy. The controlled variables included gender, age, education, employed, married, urban, health status, and household income. Robust standard errors in parentheses; *** *p* < 0.01, ** *p* < 0.05.

To verify hypothesis one and gain further insights into the role of financial literacy, we calculated separate scores for basic financial literacy and advanced financial literacy. We then conducted the same regression analysis as columns (1) and (2) in Table 3 to examine whether the basic or advanced level of financial literacy is important in the relationship. From columns (3) to (6) in Table 3, we can see that the mediating role of household indebtedness between financial literacy and mental health is only evident for basic financial literacy. In column (5), advanced financial literacy does not show any significant relationship with the household debt-to-asset ratio when covariates are controlled. Similarly, no direct relationship is observed between the level of advanced financial literacy and mental health. However, the direct relationship between basic financial literacy and mental health is evident, as seen in column (4).

Furthermore, in considering the differences between types of loans for household, we also conducted the same analysis for housing debt and non-housing debt. In view of Tables 4, 5, we can observe that there are clear differences between housing debt and non-housing debt in mediating the relationship between financial literacy and mental health. The housing debt-to-asset ratio does not show any significant mediating role for the relationship between financial literacy and mental health. It is consistent with all three measures of financial literacy we conducted, whereas the non-housing debt-to-asset ratio is significantly mediating the relationship between all three measures of financial literacy and mental health. The result in column (3) of Table 4 only shows significant negative relationship between housing debt-to-asset ratio with basic financial literacy.

**Table 4:** Mediation analysis: housing debt-to-asset ratio as a mediator.

Variables	(1)	(2)	(3)	(4)	(5)	(6)
	Housing debt	Mental health	Housing debt	Mental health	Housing debt	Mental health
Housing debt		−0.0786		−0.0722		−0.0794
		(0.0643)		(0.0641)		(0.0645)
FL	−0.00230	0.0427				
	(0.00619)	(0.0249)				
FL_basic			−0.0219**	0.137***		
			(0.0110)	(0.0457)		
FL_adv					0.0111	−0.000437
					(0.0101)	(0.0374)
Controls	Yes	Yes	Yes	Yes	Yes	Yes
Observations	3,308	3,308	3,308	3,308	3,308	3,308
R-squared	0.036	0.101	0.037	0.103	0.037	0.100

FL represents financial literacy; FL_basic represents basic financial literacy; FL_adv represents advanced financial literacy; housing debt represents housing debt-to-asset ratio. The controlled variables included gender, age, education, employed, married, urban, health status, and household income. Robust standard errors in parentheses; *** *p* < 0.01, ** *p* < 0.05.

**Table 5:** Mediation analysis: non-housing debt-to-asset ratio as a mediator.

	(1)	(2)	(3)	(4)	(5)	(6)
Variables	Non-housing debt	Mental health	Non-housing debt	Mental health	Non-housing debt	Mental health
Non-housing debt		−0.390***		−0.382***		−0.397***
		(0.0834)		(0.0831)		(0.0834)
FL	−0.0303***	0.0311				
	(0.00742)	(0.0249)				
FL_basic			−0.0619***	0.115**		
			(0.0136)	(0.0454)		
FL_adv					−0.0266**	−0.0119
					(0.0109)	(0.0374)
Controls	Yes	Yes	Yes	Yes	Yes	Yes
Observations	3,308	3,308	3,308	3,308	3,308	3,308
R-squared	0.040	0.112	0.041	0.113	0.036	0.111

*FL represents financial literacy; FL_basic represents basic financial literacy; FL_adv represents advanced financial literacy; non-housing debt represents non-housing debt-to-asset ratio*. The controlled variables included gender, age, education, employed, married, urban, health status, and household income. Robust standard errors in parentheses; *** *p* < 0.01, ** *p* < 0.05.

### 4.3 Results for hypothesis two

Table 6 presents the results of the regression analysis conducted to examine the moderating role of financial literacy in the relationship between household indebtedness and mental health.^3^ Separate regressions were executed for overall financial literacy, basic financial literacy, and advanced financial literacy, controlling for a consistent set of covariates. The findings presented in column (1) of Table 6 indicate a significant relationship between the interaction of the debt-to-asset ratio and overall financial literacy. Specifically, respondents with higher levels of debt-to-asset ratios who possess greater financial literacy scores are more likely to report better mental health outcomes. This supports our second hypothesis, suggesting that financial literacy positively moderates the nexus between household indebtedness and mental health. However, when examining the effects of basic and advanced financial literacy separately, a consistent moderating effect is observed only for basic financial literacy. As shown in column (3) of Table 6, advanced financial literacy does not significantly moderate the relationship between household indebtedness and mental health.

**Table 6:** Financial literacy as a moderator between household indebtedness and mental health.

Variables	(1)	(2)	(3)
	Mental health	Mental health	Mental health
Debt-to-asset ratio	−0.256***	−0.239***	−0.276***
	(0.0558)	(0.0546)	(0.0571)
FL	0.0336		
	(0.0248)		
FL × Debt-to-asset ratio	0.0511***		
	(0.0177)		
FL_basic		0.117***	
		(0.0452)	
FL_basic × Debt-to-asset ratio		0.106***	
		(0.0333)	
FL_adv			−0.00769
			(0.0372)
FL_adv × Debt-to-asset ratio			0.0578
			(0.0295)
Controls	Yes	Yes	Yes
Observations	3,308	3,308	3,308
R-squared	0.112	0.114	0.111

FL represents financial literacy; FL_basic represents basic financial literacy; FL_adv represents advanced financial literacy. The controlled variables included gender, age, education, employed, married, urban, health status, and household income. Robust standard errors in parentheses; *** *p* < 0.01, ** *p* < 0.05.

These findings suggest that basic financial literacy may be sufficient for managing day-to-day household finances and developing effective coping mechanisms for debt-related stress. Individuals with a solid grasp of basic financial concepts are likely better equipped to budget effectively, manage credit, and comprehend the implications of borrowing, thereby potentially mitigating the negative psychological effects of indebtedness. In contrast, advanced financial literacy appears less prevalent, with only a small proportion of respondents demonstrating proficiency. Given that advanced financial concepts are primarily relevant to investment decisions or complex financial planning, their practical utility in routine household financial management may be limited for most individuals. Consequently, it is unsurprising that advanced financial literacy does not offer comparable protective benefits against debt-related stress.

In addition, we further conducted moderation analyses specifically focusing on housing debt and non-housing debt. The results, presented in Tables 7, 8, reveal a marked difference in the moderating role of financial literacy for these two types of debt. In Table 4, no significant direct association was found between the housing debt-to-asset ratio and mental health; consequently, it is reasonable to conclude that overall financial literacy, whether assessed on a basic or advanced level, does not significantly moderate the impact of housing debt on mental health outcomes. Conversely, regarding non-housing debt, as indicated in column (2) of Table 8, only basic financial literacy demonstrates a significant positive moderating effect on the relationship between non-housing debt-to-asset ratio and mental health. Notably, overall financial literacy loses its significant moderating influence in this context.

**Table 7:** Financial literacy as a moderator between household housing debt and mental health.

Variables	(1)	(2)	(3)
	Mental health	Mental health	Mental health
Housing debt	−0.0879	−0.0752	−0.0899
	(0.0673)	(0.0649)	(0.0681)
FL	0.0428		
	(0.0249)		
FL × Housing debt	0.0242		
	(0.0237)		
FL_basic		0.138***	
		(0.0456)	
FL_basic × Housing debt		0.0410	
		(0.0451)	
FL_adv			−0.00189
			(0.0375)
FL_adv × Housing debt			0.0347
			(0.0342)
Controls	Yes	Yes	Yes
Observations	3,308	3,308	3,308
R-squared	0.101	0.103	0.101

FL represents financial literacy; FL_basic represents basic financial literacy; FL_adv represents advanced financial literacy; Housing debt represents housing debt to asset ratio. The controlled variables included gender, age, education, employed, married, urban, health status, and household income. Robust standard errors in parentheses; *** *p* < 0.01, ** *p* < 0.05.

**Table 8:** Financial literacy as a moderator between household non-housing debt and mental health.

Variables	(1)	(2)	(3)
	Mental health	Mental health	Mental health
Non-housing debt	−0.342***	−0.317***	−0.381***
	(0.0848)	(0.0804)	(0.0864)
FL	0.0322		
	(0.0249)		
FL × Non-housing debt	0.0411		
	(0.0260)		
FL_basic		0.116**	
		(0.0453)	
FL_basic × Non-housing debt		0.0993**	
		(0.0452)	
FL_adv			−0.0106
			(0.0376)
FL_adv × Non-housing debt			0.0317
			(0.0477)
Controls	Yes	Yes	Yes
Observations	3,308	3,308	3,308
R-squared	0.113	0.115	0.112

FL represents financial literacy; FL_basic represents basic financial literacy; FL_adv represents advanced financial literacy; non-housing debt represents non-housing debt-to-asset ratio. The controlled variables included gender, age, education, employed, married, urban, health status, and household income. Robust standard errors in parentheses; *** *p* < 0.01, ** *p* < 0.05.

## 5 Discussion

Economic theories show less interest in explaining the relationship between household debt and psychological problems, despite the increasing concern regarding its negative implications. The economic perspective suggests that rational economic agents, such as debtors, will optimize their decision-making in managing their debt. However, psychologists have provided empirical evidence challenging this notion, indicating that the optimal situation is not easily attainable. It is not just the financial wellbeing of households that is affected by debt; it can also impact the psychological wellbeing of the economic agent, thereby further affecting the resources available for the healthcare system (59). These challenges raise questions about how to reduce this association as the impact on wellbeing extends beyond simply reducing debt.

Therefore, this research employed nationally representative data from China to examine how financial literacy plays a role in the negative connection between household indebtedness and mental health. Our findings confirm the two hypotheses regarding how financial literacy interacts with household indebtedness and mental health. One hypothesis is that the level of financial literacy shows a negative relationship with household indebtedness and further that household indebtedness shows a negative relationship with positive mental health. The second hypothesis is that the level of financial literacy has a positive moderating effect on the relationship between household indebtedness and mental health. Moreover, considering the challenges posed by variations in financial literacy measures and their influence on empirical findings, our analysis also finds that basic financial literacy is an important factor in explaining the relationships under different model specifications. We further find that basic financial literacy plays a crucial role in the relationships for non-housing debt rather than housing debt.

Meanwhile, the importance of financial literacy and the lack of it among the population has been given increasing attention by policymakers in both developed and developing countries (61). Financial literacy is not a luxury but rather a necessity for individuals to manage their personal finances, although China has developed a comprehensive financial technology ecosystem, especially with respect to digital payments, which far surpasses other countries in the world. However, a significant number of its people remain financially illiterate (57). The fast-growing digital banking and lack of financial literacy raise increasing concern among academics and policymakers. Thus, the government of China has initiated and issued a state-level plan, namely, The Plan for Promoting the Development of Financial Inclusion (2016–2020), in this regard.

Our study provides evidence to support scholars’ suggestion that improving one’s financial literacy may be a better option to mitigate the negative implications of household debt. Compared to merely suggesting households to reduce their leverage or constrain household debt expansion, improving public financial literacy may be a good choice. By enhancing public financial knowledge, individuals can make more informed financial decisions, thus reducing the potential costs associated with financial burdens. Considering the psychological implications of debt, having higher financial knowledge among debtors can enhance their ability to make optimal choices that maximize the benefits of debt. Importantly, our results indicate that this improvement in financial knowledge does not necessarily require individuals to possess advanced financial expertise. Even a basic level of financial knowledge, including understanding basic interest rates, compounding, inflation, and numeracy, can be helpful.

Finally, it is worth noting that a multi-dimensional definition of financial literacy may have limitations as it may fail to uncover specific dimensions that contribute to improving financial outcomes (46, 60). Therefore, this study employs financial knowledge as a proxy for financial literacy, as it reflects debtors’ cognitive understanding of financial concepts. Financial behaviors and attitudes, which encompass healthy financial habits, beliefs, and attitudes, were not considered in our investigation. The complex interaction of specific components of financial literacy needs further investigation to understand the potential effects of improving financial literacy as a means to buffer the negative relationship between household debt and mental health. By considering this aspect, it is possible to examine whether components such as financial behaviors and attitudes contribute to outcomes such as lower debt levels, more secure debt arrangements, or more financially responsible households. For example, a sense of control in financial behavior or a positive perception of money and planning may lead to more secure debt management practices, which could, in turn, reduce the negative impact on psychological wellbeing.

## Data Availability

The original contributions presented in the study are included in the article/Supplementary material, further inquiries can be directed to the corresponding author.

## Footnotes

^1^The OECD definition presents the financial literacy as *“knowledge and understanding of financial concepts and risks, and the skills, motivation and confidence to apply such knowledge and understanding in order to make effective decisions across a range of financial contexts, to improve the financial well-being of individuals and society, and to enable participation in economic life.”*

^2^The debt-to-asset ratios were group into six groups as 1. No debt; 2; Less than 10%; 3, 10% to less than 30%; 4. 30% to less than 60%; 5. 60% to less than 100%; 6. 100% and more.

^3^Before we conduct the moderating analysis for all the regressions, the explanatory variables such as debt-to-asset ratio, housing debt-to-asset ratio, and non-housing debt-to-asset ratio and moderating variable such as all three financial literacies all have been centered to avoid multicollinearity problem.
