# Supplementary material for: Unpacking the role of financial literacy in the debt-mental health nexus: evidence from China
Source: Front Public Health. 2025 May 7;13:1563297. doi: 10.3389/fpubh.2025.1563297 (PMC12092232; doi:10.3389/fpubh.2025.1563297)
Supplement: Supplementary file 1 [file Data_Sheet_1.docx]

**Unpacking the Role of Financial Literacy in the Debt-Mental Health Nexus: Evidence from China**

**Appendix A: Mental health questions**

Here are some feelings or activities you may have experienced before. Please tell us how often you experienced them in the past month.

1. Almost daily; 2. Often; 3. Half of the time; 4. Sometimes; 5. Never

Q1 How often during the past 30 days did you feel so depressed that nothing could cheer you up?

Q2 How often during the past 30 days did you feel nervous?

Q3 How often during the past 30 days did you feel restless or fidgety?

Q4 How often during the past 30 days did you feel hopeless?

Q5 How often during the past 30 days did you feel that everything was an effort?

Q6 How often during the past 30 days did you feel that life was meaningless?

**Appendix B: Financial literacy questions**

***1. Basic financial knowledge***

FK1. According to your estimation, what is the annual interest rate of one-year fixed deposit in the bank?

1. Lower than 1%

2. 1–5%

3. 5–10%

4. 10% and higher

9. Don’t know [do not read out]

FK2. Suppose you have a one-year fixed deposit of 10,000 yuan and the annual interest rate is 3%. If you don’t withdraw within this period, how much money will you get on the due date?

1. 10,300 yuan

2. More than 10,300 yuan

3. Less than 10,300 yuan

9. Don’t know [do not read out]

FK3. After the due date of deposit in the above question, if you continue to save the money as one-year fixed deposit and the annual interest rate is 3%, how much money will you have in this account after one year, including the principal and interests?

1. 10,600 yuan

2. More than 10,600 yuan

3. Less than 10,600 yuan

9. Don’t know [Do not read out].

FK4. If the annual interest rate of your savings account is 3%, and the inflation rate is 5%, how many goods you can buy using your savings in this account after one year?

1. More than today

2. The same as today

3. Less than today

9. Don’t know [Do not read out].

FK5. Suppose Zhang San inherits 100,000 yuan today, and Li Si will inherit 100,000 yuan three years later. Who has a higher value of inheritance?

1. Zhang San has a higher value of inheritance

2. Li Si has a higher value of inheritance

3. They have the same value of inheritance

9. Don’t know [Do not read out]

***2 Advance financial knowledge***

FK6. In general, investments with high returns have high risks.

1. Right

5.Wrong

9. Don’t know [Do not read out]

FK7. In general, the risk of investing in one-share stock is smaller than that of investing in stock fund.

1. Right

5. Wrong

9. Don’t know [Do not read out]

FK8. Which of the following bank is responsible for making and carrying out monetary

policies?

1. Bank of China

2. Industrial and Commercial Bank of China

3. People’s Bank of China

4. China Construction Bank

9. Don’t know [Do not read out]

FK9. Generally speaking, which of the following investment has the highest risk?

1. Bank savings

2. National debts

3. Stocks

4. Capital funds

9. Don’t know [Do not read out]

FK10. If you purchase stocks of some company, that means:

1. No matter you hold these stocks for a short term or long term, you lend the money to the company anyway.

2. No matter you hold these stocks for a short term or long term, you are the stockholder of the company anyway.

3. You are the stockholder of the company when you hold these stocks for long term; and you lend the money to the company when you hold these stocks for short term.

4. None of the above is correct

9. Don’t know [Do not read out]

FK11. Which one of the following is correct in terms of describing capital fund:

1. Low-price (low unit value) capital fund has a better future performance.

2. In general, the same capital fund could be invested in several assets; for example, one capital fund can be invested into both stocks and bonds.

3. In general, capital fund can provide a guaranteed rate of return based on past performance.

4. None of the above is correct

9. Don’t know [Do not read out]

FK12. Which of the following statement is correct about a bank’s financial products?

1. Same as with other risky investments, a bank’s financial products are also possible to suffer from loss.

2. As safe as savings, bank financial products at least will not lose.

3. The expected profits of bank financial products are actual profits.

4. None of the above is correct.

9. Don’t know [Do not read out]

FK13. Which of the following statements correctly describes the core function of stock market?

1. Stock market is helpful in predicting the profits of stocks

2. Stock market increases the prices of stocks

3. Stock market helps to make matches between stock buyers and sellers

4. None of the above is correct

9. Don’t know [Do not read out]
